# Supplementary material for: Patterns of Intron Gain and Loss in Fungi
Source: PLoS Biol. 2004 Nov 30;2(12):e422. doi: 10.1371/journal.pbio.0020422 (PMC532390; doi:10.1371/journal.pbio.0020422)
Supplement: Table S1 — Also available at http://genes.mit.edu/NielsenEtAl/. (4.3 MB ZIP). [file pbio.0020422.st001.zip › NielsenEtAl/html/284.html]

AN2246.1.NCU01187.1.MG08643.1.FG05135.1


```
 CLUSTAL W (1.82) Multiple Sequence Alignments - Introns Inserted


Sequence 1: NCU01187.1	1646 aa
Sequence 2: FG05135.1	1698 aa
Sequence 3: MG08643.1	1679 aa
Sequence 4: AN2246.1	1552 aa
Alignment Length: 1799 aa
Number Identitical Residues: 405 aa
Alignment Score (without introns) 26659


MG08643.1 	MAGKQ-NTQKGPALVRKQTNNGNANLPVRGPVPTLAIPKVAATGTTTHYEQIQEEELIAL
NCU01187.1	MAWKKPAGKKKPAQQQTPKKNNEGNTTFPG----LKAPGQTPP-AKTHYQEVQESEVMVL
FG05135.1 	MAGKQPGVWKTPLTLN----NDTNDSSFPG----LQPTASPEVNHKIEYEELQQNEILAL
AN2246.1  	---MPHHHKKKTNVLKNSSPKVEKTRVFPD-----SRPIELPVVAPTNYQEIHQEEVEAL
          	         * .   .. . :      . .       .         .*:::::.*: .*

MG08643.1 	RAIYTDDFVELKDPHSAWK~KS-EPRFDIRIKAFSDEDVGVTLSVVMTATYPKSTPLLSF
NCU01187.1	QAIYGEDFTQHEAAHGAWQ0KS-EPRFDIKIKPSSDQELSVTLGVVMVATYPKTPPLLTI
FG05135.1 	EAIYGDDFVMHSETQSAWK0KT-EPHFDIRIKASKDEDFACTLSFVMTATYPKSPPLVTL
AN2246.1  	RSIYGDDFEDVQQRRSAWH0RSSEVSFRLHLRAPSNADVRLDLLVELPATYPKTCPNITA
          	.:** :**   .  :.**: :::*  * ::::. .: :.   * . : *****: * :: 

MG08643.1 	KDNENLSESTLFRLQKYLEREPKIFARKEEVMINSLVEDIREILEDAAQAKAQGRELPSL
NCU01187.1	KDDHSLRESTKFKIQKFVETQPKIYAQAEQEMIDQIVEGIRDILEEAAQKKVQGLEIPSL
FG05135.1 	KK-HDLKEVTQFKIQKFLETKPKIFAQDEQEMIDQIVEGVRDILEDAAQAKADGKHLPSL
AN2246.1  	ENLEDLRQGAQSRIRDVLQNKPRALLG--SEMIYELADSIQEILEDAAEAQAHDQDIPSL
          	:. ..* : :  :::. :: :*:      . ** .:.:.:::***:**: :... .:***

MG08643.1 	EEERAAHEAHLAHQAMEERKKEEQKRLEETQEEERIMSGQYEEEINRQRAKKLEIRRKNR
NCU01187.1	EEERAAHEAELARLAQSEKEREERKKLEESKEEERVLEDMLQEELKRQRNKAKESRKKNR
FG05135.1 	EEERERHEAYLAKLAQEKKEEEERKKMEETQEEERVMAEMLQQQIDRQKQKAKESKRRPN
AN2246.1  	EEERMVQEAAAIERAERAKEEELRKQQAASAKEELELKQLVQDAIN-KRTKALS-RRKSR
          	****  :**   . *   ::.* :*:   : :**  :    :: :. :: *  . ::: .

MG08643.1 	SGHLSPDRSVDHD---NEKIVFDQPCKLTDESGNELRFNTVKGMALVSTGPVCQVFTVRP
NCU01187.1	SHQLSPDRAPQDPGETDETLMFDQPCKITDGSGNALFFQTVIGKTVFREGPITTVYKVKP
FG05135.1 	G---NSHQELTPSTETDEMIEFDQFCNTTDKSGNTIVFKSVAGKCEPRQGPVSIVYTVRP
AN2246.1  	SSGLEAAGDTEGMARVPGAITFDPPLVITDADEGPLVFRAVYGKTLLKRVHGASTFIVRP
          	.   ..             : **     ** . . : *.:* *           .: *:*

MG08643.1 	VLSAGH-HRPSLVLKQEKLKSGGKEAGLFKKQLQSLES---QLESAKKYRHKNILEVMEF
NCU01187.1	VLSART-VRPSLALKQVEVKSHGKDSAQFKKQLQYLES---QMETLKKLRHQNLLPFLDF
FG05135.1 	VLANGQ-GSLTMALKETILRNTSKDSKEFKLQLQSLESRLQDLKTVKRIQHRHLVDVLDF
AN2246.1  	VVPESRPCAPLLVLKELSINEKGADALAFREQMRLSED---KLESLKRLRHQNLVDFYGF
          	*:.   .    :.**:  :.. . ::  *: *::  *.   .::: *: :*:::: .  *

MG08643.1 	RIDSDVCSDVGSPD--RTVSILTAYAKQGSLYDHMDVNGQDGQIGINRVRSWTRDLLDAL
NCU01187.1	RIDRGISDTDSSAPTIWTVSILTPLATKGPIEELLDLAG---HIDTNKAKIWTADLLEAL
FG05135.1 	RVQAGLA-TDPTVANAWTISVLSPLAEKGPLEELLELAG---QIEIGKVRSWTRDLLDAL
AN2246.1  	KIQTPLY-SGHSEDSTWTVFALVEHANKGSLSEFLDIVG---TVPVEMIRSWTIQLLEAL
          	:::  :     :  .  *:  *   * :*.: : ::: *    :     : ** :**:**

MG08643.1 	NFLHTNGIVHKSIHPHNVLLFRDETGAIIPKLADAGFQKELHDICSSSRAVTALRDAKSA
NCU01187.1	AFLHNNGIIHQDLHPANILLYRNEAGDIVPKITDSFYQRELHNLCTKIKTLTSSKAAKSA
FG05135.1 	NFLHNKNIAHQDIHPGNILLFREATGEIIPKISDAWYQREIHSINSHKPGLPGLHTAKSA
AN2246.1  	EYYHRHGFVHGNIHCGRVLLFRNTTGGTIVKLLSS-IEEALPDAAGNKR---LLMASKSP
          	 : * :.: * .:*  .:**:*: :*  : *: .:  :. : .             :**.

MG08643.1 	YWLPPEIAGTAKPHYTQKTDIWDFGVMFLQMVFDLEAPQRYQSPNDLKASLALSSEMLEL
NCU01187.1	YWFPPEIAGVTKPQYTQKTDVWDFGIVFLQMIFGLDVVEKYHSPSALMDSLSLSAPLEEL
FG05135.1 	YWLPPEIAGTSKPQYTFKTDIWDFGVVFVQMIFGLDVLRTYSSPKNLMESVTLSHSLHEL
AN2246.1  	FWFPPELT-QGNSSPTMKTDVWDLGIVFLQMAFGKDVLQRYTSANALADNLELSPPLHDL
          	:*:***::   :.  * ***:**:*::*:** *. :. . * *.. *  .: **  : :*

MG08643.1 	VSSFFHSEPSRRRRAFDLGSHHFLAATSAQVLMDDADSSVFTADSVSSMPQHPVPRLRHG
NCU01187.1	VSKFFKSDPKKRPRAFELSSSEFLATN-APIIDDED---IAVPGSLMPMPQMTPQRMRHD
FG05135.1 	VSRFFREDKQKRPRAFELGSSEFLATD-APVLFDDT---SALLSSSPATSFQPIPKFRRD
AN2246.1  	LQEFFRPSPKKRPTAFQLQPSEFFRVD-SPLIMRTS------ASSSMSLSRRP----RFE
          	:. **: . .:*  **:* . .*: .  : ::           .*  . .  .    *  

MG08643.1 	SMSRATAPQSRWATEWIEEGRLGKGGFGEVVKARNMVENVVYAVKKIRQRENESLTPIIS
NCU01187.1	SMS-RGPMSSRYRQDYVEEARLGKGGFGEVVRARKMIDGHLYAIKKITQRSQETLSEILK
FG05135.1 	STTHRGPSLSRYTEDFVEEGRLGKGGFGEVVKARKKLDGQIYAIKKITQRSQASLTEILK
AN2246.1  	SFSGGLPSFSRYHQDFDEAGRLGKGGFGEVVKARNKLDGRFYAVKKISHKSAAALKDTLS
          	* :   .  **:  :: * .***********:**: ::. .**:*** ::.  :*.  :.

MG08643.1 	EVSVFCRMGHPSVVKYVMAWIEDIFDGYEPSAGSVTASGVTDSESMS------GPNIEFT
NCU01187.1	EVRLLSQMNHPAVVRYYNTWLEEVPDYADTEGDTSTEGGATDVTDNSQVTISHGINIEFA
FG05135.1 	EVRLLSQLSHPAVVRYYNTWVEEITDQGDTEDDTSTG---YITEENTQGTESVGIDIQFA
AN2246.1  	EIMLLSRLNHPYVVRYFTAWLEEDCDQSDEEAISFTDGYSVGSRRSE--------EFEYS
          	*: ::.::.** **:*  :*:*:  *  : .  : * .                 :::::

MG08643.1 	TSGA--LDFVSSSRPPHIEFGFDSDNDDAHTEHGNEDGSDDSDESDESEAEDSDEESSEF
NCU01187.1	ESKSRGLDFMSSSGHPGIEYDYSSSEEEDDDEDEDDEEDESDNDSNDDAATVSGK--NHL
FG05135.1 	TSTG-GLDFISSN--AGADYGFEESDEDEDDSEEDSEQEDSSDEDDESLDEMSIH--RVV
AN2246.1  	TTGG--LDFISSSGYPNIEFVPDSDEEDAGTISTREKGSSPETFGTESGTG------KEL
          	 : .  ***:**.  .  ::  ...:::       .. .. .  . :.           .

MG08643.1 	EASGKMGRRSSRRPSMRPFSRKIMYLVMEYCEKS~TLRDLIMNNLCDDSEEIWRLFRQIL
NCU01187.1	GVTGRR-----PRRGSARPYKTVMYISMEYCEKR0TLRDLISRNLSKETAEIWRLFRQIL
FG05135.1 	SPDKERNAMFQRRARYQRSYRTILYISMEYCEKR0TLRDLIARNLYKNTAEIWRLFRQVL
AN2246.1  	SR--------VRSGSHGRPMLTALYIQMEYCEKH0TLRDLIKNGLYDDVDRSWRLFRQIL
          	                     . :*: ******  ****** ..* .:  . ******:*

MG08643.1 	EGLQHIHGFNIIHRDLKPENIFIKVAPDGTNAVKIGDFGLATAEMRAIADKNGNGSPSGI
NCU01187.1	EGLCHIHSLNIVHRDLKPENIFISSGPDGLDNVKIGDFGLATSGQLAIDRSTAS-----L
FG05135.1 	EGLAHIHGLSIVHRDLKPENIFISSSSDGIDNVKIGDFGLATSGQFSVEKSTTANT---L
AN2246.1  	DGLTHIHSNGIIHRDLKPDNIFIDAAS----NPRIGDFGLATSGQFTTAVRSSAAAD---
          	:** ***. .*:******:****. ..      :********:   :    .   :    

MG08643.1 	DSGDMTRSVGTSTYLAPEVRSGSNGVYTTKCD~MYALGIIFFEMCYPPMLGMQRAMVFDK
NCU01187.1	DASDMTRSIGTAVYVAPEVRTGGSGSYTSKVD0MYSLGVIFFEMSYPPMLGMQRAMVLEQ
FG05135.1 	ETDDMTRSIGTAYYSAPEVRSTVNGIYSTKVD0MYSLGIIFFEMCYLPIMGMQKADVLGQ
AN2246.1  	FGGNLTRSLGTTYYVAPEMKSGFAGHYNEKVD0DSEK-----------------------
          	  .::***:**: * ***:::   * *. * *                            

MG08643.1 	LRQPPPTLPDDFRPGEKNQKQIVSSLVDHTPSQRPSATELLKSGLLPVGMESDAMRQVMA
NCU01187.1	LRQSPPILPADFKHMDKNHLEVLLSLLTHNPKERPSSSELMKSGKLPIQMESEAIRRAIA
FG05135.1 	LRRPQPVLPSDFKPAEKAQTEIVLSLVNHNPKERPSSTDLLKSGKLPVQMESETIRRTLA
AN2246.1  	----------------VVQGEIIKSLLSHDPAERPSASELLHSGQIPLQVEEETFRRAIM
          	                  : ::: **: * * :***:::*::** :*: :*.:::*:.: 

MG08643.1 	GLE-DPNNPHYTDYISALFKRRLDRAKDYAWDAES-VNKSPAELMRLLVTEETLTAVFRR
NCU01187.1	GLS-DPNSPYYQKMLETLFSRPIEQAKDYAWDMSS-SGPSPQELMRQFIVKDTLISIFRR
FG05135.1 	GLA-DPSSPYYRKMLSTLFAKRMEPAKNYAWDMSS-ANMSPQELLNQGLVKQELVSIFRR
AN2246.1  	HLLSDPSSPDYKKILSAIFSQSPKKVEDIAWDMHSRAPPAANELLMHGLVKERLTSIFRK
          	 * :**..* * . :.::* :  . .:: ***  *    :. **:   :.:: * ::**:

MG08643.1 	HGAVKVSISPFYPPSAHYTQNTVEVVDKHGALLQLPYDLTMGSARALAKSTSRDTVVQRS
NCU01187.1	HGAVEAPTVCLYPSSSHYGQNAVHLLDQNGTVLQLPFDLMMGHARSLARITN-SPVVQKS
FG05135.1 	HGALETPRGIIYPKSTHYGDNAVQLLDPNGNVLQLPYDLTLGNARIMAKQTN-GPVVQRT
AN2246.1  	HGAVETTRQMLFPKSQHYNSGDVRLLDASGNMLQLPFDLTLPNARAIPRQDP---SLEKT
          	***::..   ::* * ** .. *.::*  * :****:** :  ** :.:       ::::

MG08643.1 	YSFGRVFRDRPSGGQPQMFGEVHFDVVSEDASDLALKEAEAIKVLDEIIAEFPTLSRSQM
NCU01187.1	YSFGNIFRDRHGGGQPDVYGEVDFDIVTSDALDLALKEAEVIKVLDEIATAFPTVSSTPI
FG05135.1 	FTFGNVFRDKQDMGQPLMFGEVDFDIVTTDTLDLALKEAEVLKVIDEIIHTFPSLSTTAM
AN2246.1  	FAFGTVYRDTPHGGEPRTHKEVDFDIVSRNTLDLALKEAEVIKVLDEIIEEFPPLKASAM
          	::** ::**    *:*  . **.**:*: :: ********.:**:***   **.:. : :

MG08643.1 	CFHLGHSDLLRCIFDYCGVEKASRRAVADTLSKLNIRGVTWQKIKSELRT-TASISTTTL
NCU01187.1	CFQLGHSDLLNLIFEYCGVEVGTRRAAAEVLSKLNIRNFTWQKVRGELRSPMVGISATSV
FG05135.1 	CFHLGHSDLLHLIFEHCGIEPASRSGAADVLSRLNIHNYTWQKIRIELRSPTVGVSATSV
AN2246.1  	CFLINHSDLLQLVMEFCRIKPSQIPLVKEVISRLNVGKYSMQKIRSELRSPTIGVASTSL
          	** :.*****. :::.* :: .    . :.:*:**:   : **:: ***:.  .:::*::

MG08643.1 	DELQKFDFRE~NPGKAANQLRTIFQKTDVSQQVSQPLAHLKEVWEYCKKLGVRTKIYVAP
NCU01187.1	DELQRFDFRD1TPSKAIAKIKLLFEGTEYYQQASSTLAHLKEVYEYTKRFGVQNKIYIAP
FG05135.1 	DELQKFDFRD1TPNKTFAKLKTLFEGSDMYQRASPTIAHLKEVVEYCKHLSVGTKVYINP
AN2246.1  	DELARFDFRD1SPKQTQKRLRAIMEGTEFAERLTPIFARINSLVTFLQGFKIKRKFFICP
          	*** :****: .* ::  ::: ::: ::  :: :  :*:::.:  : : : :  *.:: *

MG08643.1 	LWSWNEVFFSGNMMFACLFDKRSREVLAAGGRYDSLIKEHRPRISGSHFQERHAVGFGMN
NCU01187.1	LSSINEAFFRGGILFSCLYDKKVKDVFAAGGRYDSLIKEHRPKIG-NRFEERHAVGFSLN
FG05135.1 	LNSLKEAFYTGGVLFSCLYDKKVKDVFAAGGRYDQLIKEHRPKVG-SQFGERHAVGFSLA
AN2246.1  	LSSLNEKFFRGSILFQCIFDGKRRDVFAAGGRYDSLVQDFRSGVTTNRVQP-HAVGFNLS
          	* * :* *: *.::* *::* : ::*:*******.*:::.*. :  .:.   *****.: 

MG08643.1 	WEQ---LAHVSAKSSKK---RAGAADEETNLFATKR~CDVLIASFDPSLLRTTGLELLQT
NCU01187.1	WEKQ--LAKPVPKATGKTFLKKAAEEESQGLFNIKR~CDVLVASFDPEVLRSSGIELVQT
FG05135.1 	WER---LAKP-PKSGGRPFLKKAEDE-SNSIFNTRR0CDCLVASFDAAVLRSSGVEILQT
AN2246.1  	WDRLSSAMIDYVKAPTKASLKHPETD-VGAFWKTRR0CDVLVASFDPTILREMGVQLTQD
          	*:: ::      *:  :.  :    :    ::  :* ** *:****. :**  *::: * 

MG08643.1 	LWAHGISAELARDARSPEEVLQAQREDGHNWVVIVKQD--MLKIKTPGRREAPDADVLPG
NCU01187.1	LWAHSISAELARDARSPEDLLSKYRDESYLWVVIIKQDN-MLKIKSMARKDAPDADIPAK
FG05135.1 	LWAHDISAEMAKDARSPEDLMSKHRDETYSWIIIIKQDS-ILKIKSMGRKDVPDADMPST
AN2246.1  	LWSNSISAELAVDASSLEELLTKYKEHNHSWIVIAKQDSKERGFKVRCLVPKEELDIRGS
          	**::.****:* ** * *:::   ::. : *::* ***..   :*        : *:   

MG08643.1 	QLVQWLRAEMREREGRIVQASRLRATAGGDGNAAAVVGSYGGYSYHGRHNDYMAGPGIMS
NCU01187.1	ELLNWLKAEMRENRD-----ALMRGTGSGSISSGAAIKFRGGGGSGGGGGLSSFGTNNSE
FG05135.1 	QLLAWLRNEIRER------------------DSRSVVKLRG--------------NSTTD
AN2246.1  	ELIPWLRSEIRARNQ----------REGAPENPRFAR-----------------LPSQSE
          	:*: **: *:* .              ..  ..  .                    .  .

MG08643.1 	SGGGVISPGTDSLTVHPHREQEVRVITARTRSKKSNRQALVEQA~QAAAARVASTMVRFA
NCU01187.1	LNSGLF------LGADGEREQEVHVLVAQTKSKKFNRRQVVEQA~QMSAAKLLQSFL---
FG05135.1 	SNS-----------GDKESEQEVRVLVAQTRSKKFNRRTVVEQA~QVSASSLVRSFL---
AN2246.1  	VNT-----------PANDRSNDVRILVSQHRSKKTNRRNIVEQA1LTRAREVVENALN--
          	 .               . .::*:::.:: :*** **: :****    *  :  . :   

MG08643.1 	SDANSTGPVVAIETTDAVMSLIQSTRLSDAESWRRAEQAVGMEERQYVRELRGLLQDMRA
NCU01187.1	-----DGPIAAIETSDSVMDMIRRTSLSDAESWRRVEHNVGTSEKKYVKEIHDMLKGWRW
FG05135.1 	-----EGPVLAIETTDQVMDLMRETCLSEPDGWRQVEHSVTNTEKKYIREIHDQLDTWRD
AN2246.1  	------GPIAAIDTRDDLLEAIRDTRLSDPDSWRAVIQNAPLTERKYLQQVYDLLAEFAS
          	      **: **:* * ::. :: * **:.:.** . : .   *::*:::: . *     

MG08643.1 	DQGGVGRGISGLAWSMPAFVYNFRTGYCIYYDLG~A------------~-----------
NCU01187.1	EWEAKKG-------SEHAFVYNFRTGRCIYYDLS~A------------~-----------
FG05135.1 	TYKKKNG-------SRHAFLYNFRSGNCVYYDLD1QQHSDNNALADLK0INVVSTYCADI
AN2246.1  	ESHGDSAD-----YPGNAFIYNFRTGSCLYYDLG~SANER--------~-----------
          	       .      .  **:****:* *:****.    .                     

MG08643.1 	-----------------~-------------~----------------------------
NCU01187.1	-----------------~-------------~----------------------------
FG05135.1 	AALTLELKQVTTVCFGF1NIPRHPSDREQTV1FSTEAGTRRGKAGANRACVSVRHGMMAA
AN2246.1  	-----------------~-------------~----------------------------
          	                                                            

MG08643.1 	~---------
NCU01187.1	~---------
FG05135.1 	1YPIVLSVCE
AN2246.1  	~---------
          	
```
